# Nano-radiopharmaceuticals as therapeutic agents

**DOI:** 10.3389/fmed.2024.1355058

**Published:** 2024-03-15

**Authors:** Tanu Dixit, Nayomi Dave, Kausani Basu, Pranav Sonawane, Trutuja Gawas, Selvan Ravindran

**Affiliations:** Symbiosis School of Biological Sciences, Faculty of Medical and Health Sciences, Symbiosis International (Deemed University), Lavale, Pune, India

**Keywords:** nanotechnology, radiopharmaceuticals, nuclear medicine, nanoradiopharmaceuticals, nanomedicine, biodistribution, drug delivery systems

## Abstract

In recent years, there has been an increased interest in exploring the potential synergy between nanotechnology and nuclear medicine. The application of radioactive isotopes, commonly referred to as radiopharmaceuticals, is recognized in nuclear medicine for diagnosing and treating various diseases. Unlike conventional pharmaceutical agents, radiopharmaceuticals are designed to work without any pharmacological impact on the body. Nevertheless, the radiation dosage employed in radiopharmaceuticals is often sufficiently high to elicit adverse effects associated with radiation exposure. Exploiting their capacity for selective accumulation on specific organ targets, radiopharmaceuticals have utility in treating diverse disorders. The incorporation of nanosystems may additionally augment the targeting capability of radiopharmaceuticals, leveraging their distinct pharmacokinetic characteristics. Conversely, radionuclides could be used in research to assess nanosystems pharmacologically. However, more investigation is needed to verify the safety and effectiveness of radiopharmaceutical applications mediated by nanosystems. The use of nano-radiopharmaceuticals as therapeutic agents to treat various illnesses and disorders is majorly covered in this review. The targeted approach to cancer therapy and various types of nanotools for nano-radiopharmaceutical delivery, is also covered in this article.

## Introduction

Numerous commonly accessible radiopharmaceuticals are used for illustrating the structure and operation of bodily tissues, organs, and cells. These radiopharmaceuticals are designed to treat a variety of cancers, joint problems, pain relief from bony metastases, and numerous other conditions of a similar nature. Essentially, nuclear medicine constitutes a medical discipline that employs radiotracers and carrier molecules to visualize the local biochemistry of the body. Impacts on organ absorption, retention, transportation, and biological distribution toward the targeted location depends on the biochemical properties of the carrier molecule and radiotracer. Thus, for a deeper understanding, it is imperative to comprehend the biochemistry of radiopharmaceuticals (1). Nuclear pharmacists need to be aware of the action process, or how radiopharmaceuticals localize and start working. This knowledge is crucial to evaluate the pharmacokinetics and substrate specificity of the labeled medication.

According to Vallabhajosula et al., radiopharmaceuticals (2) offer us the chance to do prompt diagnostics utilizing blood flow, multimolecular cellular localization, bioenergies, tissue metabolism, the physiological activity of the specific organ, and intercellular and intracellular signaling networks. Depending on how each organ functions, distinct radiopharmaceuticals are utilized to scan different organs. Because inorganic iodine is more readily absorbed in the thyroid, labeled iodine, for instance, would be perfect for imaging thyroid cancers. In a similar vein, radiolabeled phosphate is frequently utilized for bone scans because it has been found that phosphate ions accumulate greater in bone. As a result, since tagged atoms are more concentrated in organs, they can be used for organ imaging. Numerous radiopharmaceutical agents in various phases of clinical development or already commercialized are presented in Table 1.

**Table 1:** Radiopharmaceuticals in various clinical development phases.

Radiopharmaceutical	Disease/disorder	Company name	Development phase/ commercial	Clinical phase identification number	References
^177^Lu-labeled PSMA-617	Metastatic Castration-Resistant Prostate Cancer	Endocyte	Phase 3	NCT03511664	(3)
^177^Lu-labeled NeoBOMB1	GRPR over expressing tumor	Novartis	Phase I/IIa	NCT03872778	(4)
^166^Ho microspheres	Unresectable Hepatocellular Carcinoma	Marnix Lam, UMC, Utrecht	Early Phase II Study	NCT05114148	(5)
^166^Ho microspheres	Neuroendocrine Tumors	Terumo	–	NCT02067988	(6)
^177^Lu-labeled PSMA-R2	Metastatic castration-resistant prostate cancer (mCRPC)	Advanced Accelerator Applications	Phase 1/2 study	NCT03490838	(7)
*^225^Ac*-*labeled aCD38*	Multiple myeloma	Actinium Pharmaceuticals	Open label Phase I trial	NCT02998047	(8)
*^177^Lu-labeled CTT-1403*	Prostrate cancer	Cancer Targeted Technology	Phase 1 clinical trial	NCT03822871	(8)
*^227^* conjugate *PSMA*-*TTC*	Metastatic Castration Resistant Prostate Cancer (mCRPC)	Bayer	Phase 1	NCT03724747	(9)
^227^Th-labeled aCD22-TTC (BAY 1862864)	Lymphoma, non-Hodgkin	Bayer	Open-label Phase I	NCT02581878	(10)
^227^Th-labeled MSLN-TTC	Mesothelin tumor	Bayer	Phase I/II	NCT03507452	(11)
^225^Ac-labeled FPX-01a	Lung cancer	J&J / Fusion Pharma	Phase I	NCT03746431	(8, 12)
Radium-223 chloride^a^	Bone metastasis	Bayer	Commercialized	–	(13)

a-alpha emitter based radiopharmaceutical therapy (RPT) agents.

There is an abundance of mechanisms via which radiation therapy affects cancer cells. The principal applications of high-energy ionizing radiations, such as gamma rays and X-rays, involve ionizing water or biological components. In selective scenarios, particulate radiations like electron, proton, or neutron beams and alpha or beta particles are utilized to target cancerous tissues. As a major constituent of cellular composition, water is the principal target for ionizing radiations. These radiations induce the lysis of water molecules through radiolysis, resulting in the generation of charged species and free radicals, including hydroxyl radicals (OH^•^), hydrogen radicals (H^•^), superoxides (O_2_^−^), and charged water species such as H_2_O^+^ and H_2_O^+^. In contrast to chemical lysis, this radiolysis process occurs due to the impact of radiation. Although many other biological components are harmed, DNA is the main target of ionizing radiation and radicals. Free radical interaction with cell membrane structures also results in structural damage that triggers apoptosis. The hydroxyl ion is well-documented as a primary initiator of lipid peroxidation and cellular damage. Empirical evidence has illustrated that interaction with lipid bilayers enhances cellular permeability (14).

Medical and scientific research has garnered much interest in nanomaterials (15). In their early iterations, significant amounts of drug were first delivered using nanoparticles as delivery agents. Subsequently, radionuclides were used to tag nanomaterials to investigate *in-vivo* biodistribution, pharmacokinetics, and pharmacodynamics. Nanomaterials attached to radionuclides have become increasingly promising for cancer treatment. Common characteristics of these formulations include high surface area-to-volume ratios, efficient radionuclide loading and labeling, and straightforward synthesis, enabling the production of constructs with diverse physico-chemical characteristics, shapes, and sizes.

Nanomaterials have a few other characteristics in addition to the ones stated above that may make them appealing for use in medicine. One of these is the ability to easily build nanotheranostics by multiplexing therapeutic and diagnostic radionuclides onto the same nanomaterial framework. Nanomaterials can be customized with vectors with homing components specifically designed to bind to overexpressed receptors on tumor cells. This enables the radiolabeled nanomaterial with several functions to engage with the target site via various receptors, resulting in increased selectivity and accumulation at higher concentrations (16). In addition to these characteristics, nanomaterials may be able to lower associated side effects and boost specificity (17). In principle, nanomaterial-based formulations can potentially augment both the conventional imaging capabilities and therapeutic efficacy of radionuclides. Additionally, they can be readily tailored to address specific limitations inherent in traditional radionuclide therapy. Based on these potentialities of nanomaterials, the present review focuses on using nano-radiopharmaceuticals as therapeutic agents for treating various diseases/disorders.

## Targeted approach for cancer therapy employing radiopharmaceuticals

**Figure 1:**
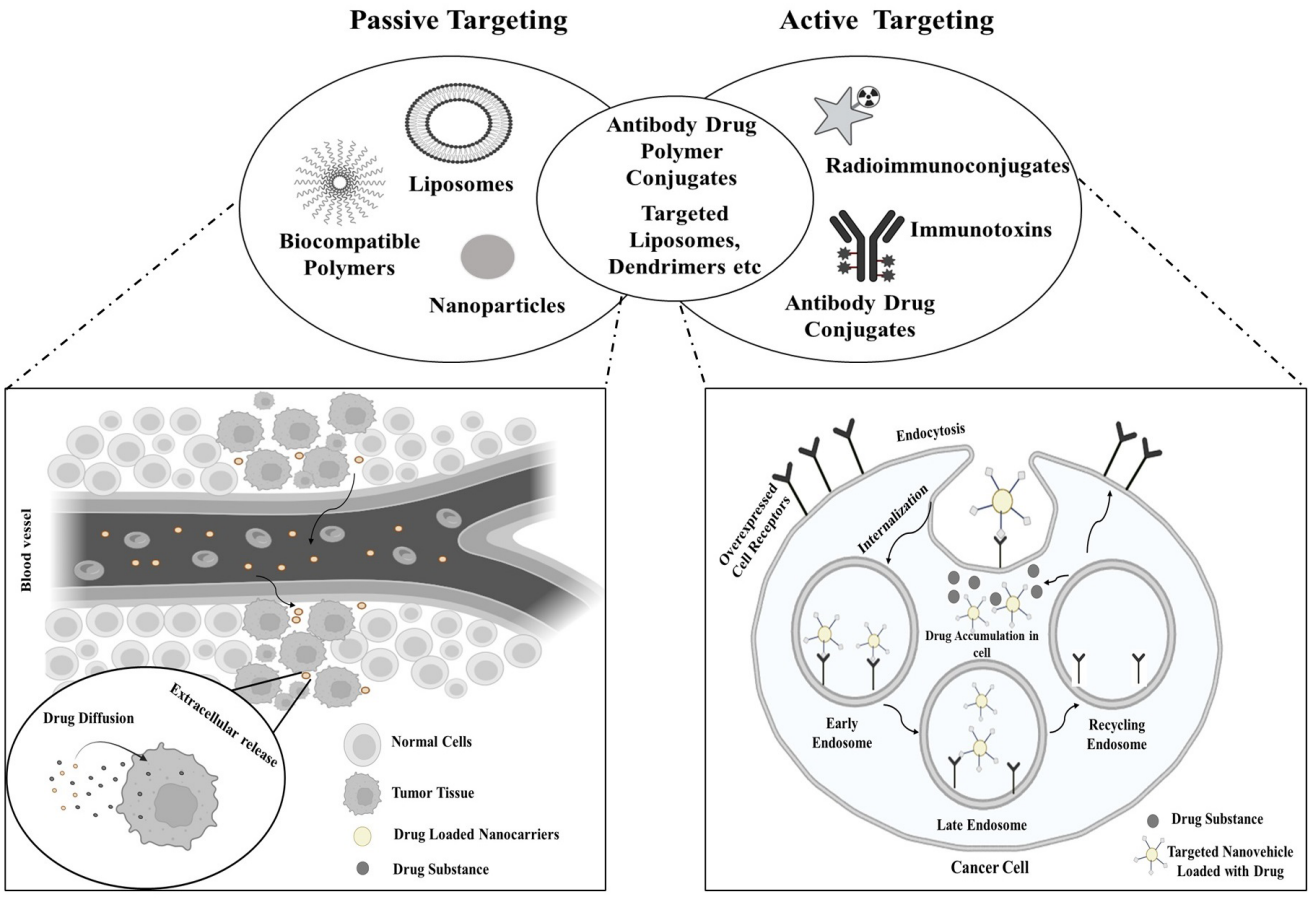
Targeting approach of radiopharmaceuticals in cancer therapy.

Exploiting specific characteristics of tumors, such as angiogenesis and the distinct tumor microenvironment that differentiates it from the vasculature and surroundings of normal tissues, intravenously administered nanostructures exhibit a notable predilection for tumor accumulation over normal tissues (18). This is referred to as the EPR effect, a physiological phenomenon characterized by increased absorption and decreased clearance, permits passive buildup of nanostructures in tumors without causing comparable accumulation levels in nearby normal tissues (19). The targeting approach employing radiopharmaceuticals to target cancer cells is illustrated in Figure 1.

Passive targeting encompasses the infiltration of the targeting agent into the tumor site via permeable blood vessels and other components generated by the tumor. This involves the agent traversing the bloodstream to reach the tumor site and subsequently accruing at the tumor site due to insufficient drainage mechanisms. The effectiveness of the radiopharmaceutical in this form of targeting is dependent on the circulation time. For passive targeting, a variety of polymer-radiopharmaceutical combinations have been studied (20). The biological interaction of radiopharmaceutical agents with tumor surfaces is known as active targeting. In active targeting, the radionuclide is conjugated to tumor-specific vectors (such as peptides or antibodies), with or without the incorporation of chelators. Tumors with poor permeability are most suited for active targeting, which identifies certain cells within the tumor milieu (21).

## Types of nanotools for the delivery of nano radiopharmaceuticals

**Figure 2:**
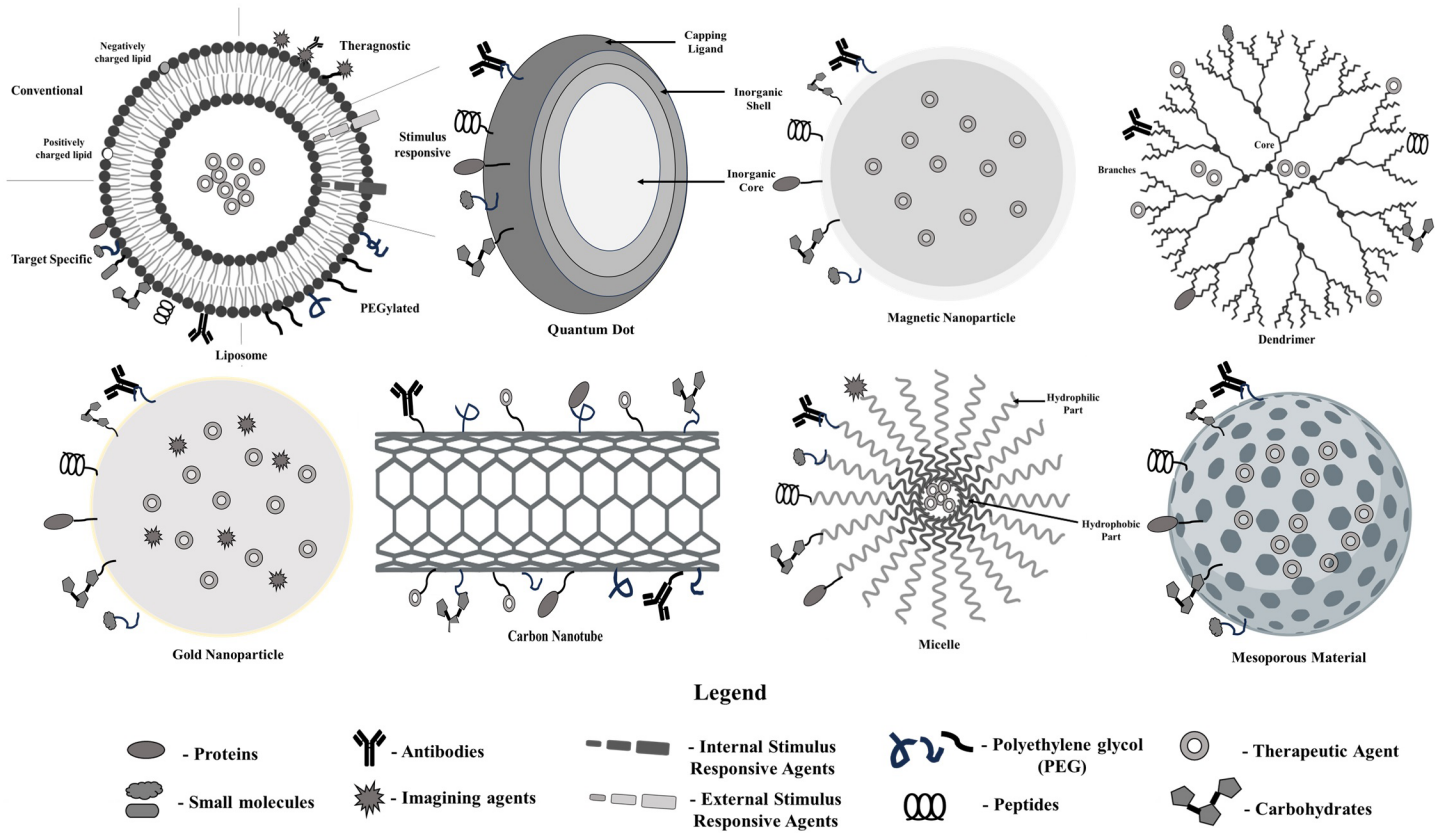
Nanotools for the delivery of pharmaceuticals.

An array of nanotools have been widely investigated in the past years for the delivery of therapeutics in order to treat a number of diseases (Figure 2). The following section enlists a handful of nanotools for the delivery of nano radiopharmaceuticals.

- Polymeric nanoparticles

Polymeric nanoparticles (NPs) are solid colloidal particles that exhibit distinct characteristics like increased surface-to-volume ratio, biodegradability, quantum properties, low cytotoxicity, and the capacity to adsorb and transport additional molecules (22–24). Furthermore, NPs are solid in nature. One of the most crucial problems in pharmaceuticals is the utilization of polymeric nanoparticles in the drug delivery sector.

About 90% of nuclear medicine diagnostic procedures utilize ^99^mTc (25). Nanoradiopharmaceuticals based on ^99^mTc and, more recently, rhenium-186 have become indispensable tools for detecting and treating various illnesses or malfunctions in the body’s organs and systems (25, 26). The emergence of these nano radiopharmaceuticals provides an appealing alternative for tumor treatment and diagnosis, introducing a novel approach to nuclear medicine, radioprotection, and dosimetry (27). Other radiopolymers such as rhenium-186 etidronate, samarium-153 lexidronam, and strontium-89 chloride are currently employed to alleviate bone discomfort associated with bone metastases.

Despite variations in several aspects among these radiopharmaceuticals, no documented benefit in terms of a higher response rate has been observed, including physical half-life, beta energy, penetration spectrum, and biochemical characteristics (28). Technetium-99 m is the most widely employed SPECT radionuclide due to its optimal imaging properties, such as a 140 keV γ emission and a short half-life of 6.0 h. In exploring the biodistribution features of nanoparticles (NPs), ^99^mTc has been utilized for a more in-depth understanding. In an effort to traverse the blood–brain barrier (BBB), Bikhezar et al. explored (29) the utilization of polymeric nanocarriers encapsulating MEK162 (binimetinib, a MEK1/2 inhibitor). The *in vitro* model demonstrated the effective penetration of the nanosystem through the blood–brain barrier, suggesting its capability to transport therapeutic medications to brain tumor locations. Furthermore, it exhibited efficacy in suppressing tumor growth when employed in conjunction with temozolomide (TMZ) and radiation therapy (RT) for treating glioma spheroids (29). Ozgur et al. (30) investigated the radiopharmaceutical potential of pheophorbide, utilizing ^99^mTc-labeled bovine serum albumin nanoparticles. These nanoparticles demonstrate promise for application in scintigraphic tumor imaging and drug delivery, as evidenced by their heightened uptake in breast and uterine tissues compared to ^99^mTc-labeled pheophorbide-a (30).

Liposomes and micelles derivatized with diethylenetriamine pentaacetic acid (DTPA) have been demonstrated to encapsulate radiolabeled ^111^In and ^177^Lu. The utilization of ^111^In-labeled NPs has been prevalent in assessing the biodistribution of NPs. Notably, these NPs exhibited substantial aggregation in the liver and spleen of healthy Lewis rats 12 h post-injection, with minimal intestinal excretion. Importantly, the complexes retained high radioactivity concentration, indicating minimal release of metals (31, 32). Additionally, studies have shown that ^111^In-labeled gold nanoparticles effectively target αvß3 integrin both *in vivo* and *in vitro* using human melanoma and glioblastoma models. Another investigation revealed that a ruthenium-based radiosensitizer, combined with ^111^In-labeled polymeric nanoparticles, may induce combinational and targeted therapeutic effects on cancer cells overexpressing the human epidermal growth factor receptor (EGFR) (33, 34).

- Lipid based nanoparticles

Liposomes, characterized by a lipid bilayer structure, serve as drug delivery systems with a hydrophilic interior capable of incorporating radiopharmaceutical agents. Various techniques have been employed for radiolabeling liposomes, including the use of lipophilic chelators like 2-hydroxyquinoline with preformed liposomes to load radionuclides via ionophores. Other approaches involve employing DOTA chelators or PEGylation for surface labeling onto the lipid bilayer, as well as using chelators like DOTA for the passive encapsulation of radioisotopes or membrane labeling during the preparation process (35).

^111^In ^111^In-labeled vinca alkaloid, namely vinorelbine is an antiproliferative agent that has demonstrated reduced toxicity and improved tumor reduction in mouse models. Vinorelbine is available in liposomal formulation. Because of their dual energy release characteristics that can be used to both impact cell death and for imaging, rhenium isotopes are especially well-suited for theranostic purposes. As demonstrated in a mouse model of head and neck cancer, the concurrent administration of other cytotoxic medications, such as doxorubicin, can increase the potency of such radioisotopes. Chang et al. (36) developed a liposome formulation by combining ^188^Re with sorafenib.

Liposomes conjugated with vasoactive intestinal peptide have exhibited enhanced accumulation of encapsulated Tc-HMPAO (hexamethylpropyleneamine oxime) in a breast cancer model. Furthermore, liposomes created with pertechnetate and surface charge modifications have demonstrated the ability to evade the reticuloendothelial system (37). Comparable intratumoural accumulation of Tc-liposome and Doxil liposomal formulations serves as confirmation of the said approach. Developing ^67^Ga liposomes with specific surface charges that allow for *in vivo* tumor and inflammation differentiation is also possible. ^64^Cu has also been shown to improve *in vivo* tumor accumulation in neuro-endocrine, head, and neck, and breast malignancies (38). Because 1-[^18^F]fluoro-3,6-dioxatetracosane penetrates the blood brain barrier differently than ^18^F-deoxyfluoroglucose, liposomes containing it function better for *in vivo* neuroglioma imaging.

Patients undergoing combination therapy involving a liposome formulation and another chemotherapeutic, such as cisplatin, have exhibited positive correlations between patient outcomes and the intratumoral retention of radiotagged liposomal anticancer formulations. Medication-free radiotagged liposome diagnostic companion kits have advanced because of these results, despite the increased drug concentration inside the tumor, which does not always translate into increased efficacy. Doxil^TM^’s comparable effectiveness to free doxorubicin across a range of cancer types is an example of the abovementioned concept. However, liposomal Doxil^TM^ reduces cardiac tissue toxicity and lowers dosage frequency (39).

*In vitro* characterization studies, including assessments of particle size, zeta potential, and high-performance liquid chromatography (HPLC), were conducted alongside *in vivo* toxicity tests. These investigations utilized ^99^mTc-labeled cationic PEGylated liposomes produced through conventional thin-film hydrolysis (40). The outcome illustrated that adding free liposomes utilizing a pH gradient approach improved the radiotracer’s uptake and localization. Even greater specificity was demonstrated, nevertheless, by tracer encapsulation that occurred during liposome synthesis.

- Quantum dots

Quantum Dots (QDs) have emerged as potent tools for drug delivery in various scientific fields, including molecular biology, cell biology, molecular imaging, and medical diagnostics. These QDs have been extensively investigated under diverse conditions, both in cells and living animals, primarily for imaging purposes. Apart from non-specific QD distribution/accumulation usage such lymph node mapping, vascular imaging, etc. (41), several research groups have also succeeded in active tumor targeting with QD-based probes (42).

Dynamically radio-labeled Quantum Dots (r-QDs), incorporating ^109^Cd into the core/shell structure of QDs with various compositions, were synthesized. The *in vitro* and *in vivo* characterization of these r-QDs was subsequently investigated (43). The near-infrared emission, extended circulation half-life, minimal cytotoxicity, tiny dimensions, and low accumulation in the reticuloendothelial system, and precision in measuring their biodistribution in mice were the intriguing features of these r-QDs. The study showcased the desirable properties of intrinsically radio-labeled Quantum Dots (QDs), suggesting that their biological potential could be further enhanced through ongoing development and optimization.

One research employed antibody-conjugated Cd^125m^Te/ZnS QDs to target the pulmonary endothelium of mice. Biodistribution investigations and SPECT imaging were used to assess the targeting efficacy, although no optical imaging was described (44). Functionalized Cd^125m^Te/ZnS QDs interact with the reticuloendothelial system, and the potential advantages of vascular targeting were investigated (45). The study revealed a consistent radioactive distribution in the mouse lungs, with notably lower accumulation in the liver and spleen than non-targeted Cd^125m^Te/ZnS QDs. This indicated the antigen-specific absorption of antibody-conjugated Cd^125m^Te/ZnS QDs. Biodistribution tests were also conducted in animals with depleted phagocytic cells using clodronate-loaded liposomes, revealing a significant reduction in QD absorption and elimination from the circulation.

- Mesoporous nanoparticles

Because of their high capacity, ease of breakdown, and low toxicity, mesoporous nanomaterials have proven to be good drug carriers (46). For example, mesoporous tantalum oxide (mTa_2_O_5_) nanoparticles were modified by Chen et al. using polyethylene glycol (PEG) to create novel nanocomposites (mTa_2_O_5_-PEG). Chemotherapy medications like doxorubicin (DOX) can be efficiently loaded and delivered by these mTa_2_O_5_-PEG nanocomposite materials. Ta, a high Z element, can boost radiation’s anti-tumor effects and increase the amount of X-rays deposited within tumor tissues in mTa_2_O_5_-PEG/DOX nanoparticles. In comparison to free DOX administered at a similar dose in conjunction with radiotherapy (RT) *in vivo*, the toxicity of DOX-loaded mTa_2_O_5_-PEG nanoparticles combined with RT was significantly reduced (15). In a separate study by Liu et al., bi-based mesoporous litchi-shaped Na_0.2_Bi_0.8_O_0.35_F_1.91_:20%Yb nanoparticles were loaded into amphiphilic polyethylene glycol (PEG) as a drug delivery vehicle. This nanocomposite exhibited controlled release of chemotherapy medication like DOX. According to Liu et al. (47), NBOF-PEG nanoparticles containing the high-Z element Bi, can potentially enhance the anti-tumor efficacy of radiotherapy by increasing X-ray absorption in tumor tissues.

## Conclusion

Collaborative research at the nanotechnology-nuclear medicine interface offers potential solutions to current challenges in radionuclide therapy. Cross-training scientists across disciplines is essential for sustained multidisciplinary growth, fostering innovative findings and translational opportunities. This research highlights distinct advantages and limitations in imaging modalities, nanomaterial radiolabeling, and nano-radiopharmaceutical compositions. Despite incremental improvements, challenges persist, including precise radiation dose selection, reliance on costly noble metals, and the long-term toxicity of accumulated nanoparticles. A holistic strategy, which balances drawbacks and benefits, necessitates multidisciplinary efforts at the convergence of nuclear medicine and nanomedicine. This approach holds promise for tailored solutions, facilitating the seamless transition of novel agents from bench to bedside soon. Striking a balance between potential drawbacks, such as elimination kinetics and manufacturing/regulatory challenges, and the evident benefits of nano-radiopharmaceuticals in loading efficiency and therapeutic efficacy is crucial. Anticipating future trends and promising preclinical outcomes, we foresee a prospective landscape where novel agents seamlessly transition from bench to bedside. This collaborative endeavor, driven by multidisciplinary experts, is pivotal for advancing convergent research and realizing the full potential of this intersection.

## Author contributions

TD: Data curation, Software, Writing – review & editing. ND: Methodology, Writing – review & editing. KB: Formal analysis, Investigation, Writing – review & editing. PS: Validation, Writing – review & editing. TG: Formal analysis, Writing – review & editing. SR: Conceptualization, Writing – original draft.
